# Editorial: Women in radionuclide therapy: 2023

**DOI:** 10.3389/fnume.2024.1411878

**Published:** 2024-05-09

**Authors:** Egesta Lopci, Federica Matteucci

**Affiliations:** ^1^ Nuclear Medicine Unit, IRCCS-Humanitas Research Hospital, Rozzano, Italy; ^2^ Nuclear Medicine Operative Unit, IRCCS Istituto Romagnolo per lo Studio dei Tumori (IRST) “Dino Amadori”, Meldola, Italy

**Keywords:** radionuclide therapy, women, PRRT (peptide receptor radionuclide therapy), radioablative iodine therapy, theranostic application

**Editorial on the Research Topic** Women in radionuclide therapy: 2023

Radionuclide therapy has a rich history dating back to the early 20th century when pioneering women like Marie Curie made groundbreaking discoveries in the field of radioactivity. Since then, women have continued to contribute, often quietly, to the development and application of innovative treatments in healthcare (1). In particular, women have played a significant role in the clinical implementation of radionuclide therapy, a specialized area of medicine that utilizes radioactive substances for therapeutic purposes (2). Radionuclide therapy has been a crucial advance in the treatment of various conditions. Recent research has highlighted the effectiveness of radionuclide therapy in treating cancer patients, particularly those with advanced, metastatic disease (3, 4). Studies have shown that it can offer a survival benefit over conventional treatments, with promising long-term outcomes observed in both men and women.

In the present Research Topic, we aimed to shed light on the involvement of women in radionuclide therapy and the impact of this innovative approach on patient outcomes by promoting the publication of articles written and led by eminent female scientists. In the following paragraphs, each contribution will be separately discussed and its impact will be illustrated for the audience.

First, Salmont Higuchi et al. assessed the clinical outcome of patients with intermediate-high-risk thyroid cancer treated with radioiodine (RAI) therapy. The authors specifically wanted to compare the difference in terms of death rate, overall survival (OS), and progression-free survival (PFS) between 81 patients with differentiated thyroid carcinoma (DTC) treated with RAI, who had undergone two separate protocols for increasing serum thyrotropin levels, either by thyroid hormone withdrawal (THW) or by administration of recombinant human TSH (rhTSH). The results of this retrospective study, after a median follow-up time of 83 months, demonstrated no statistically significant difference in the above clinical outcomes. This means that there is no inferiority of one protocol over the other, with no additional risk of recurrence at long-term follow-up.

A dedicated case report was also presented by Hadad et al. on a patient with metastatic follicular thyroid carcinoma. Herein, a new re-differentiation strategy was questioned. Specifically, the authors described the history of a female patient who underwent two cycles of RAI, leading to a negative whole-body radioiodine scan (WBIS), but positive [^18^F]-FDG and [^68^Ga]-DOTATATE PET/CT at the level of metastatic lesions. Consequently, the patient had undergone four cycles of peptide receptor radionuclide therapy (PRRT), which appeared to promote the re-differentiation of all pulmonary nodules as well as bone metastases. The intriguing hypothesis reported here has not been previously documented in the literature and therefore needs to be elucidated in future studies.

PRRT is now a consolidated treatment for advanced, metastatic, or inoperable neuroendocrine tumors (NETs), particularly those that are progressive and traditionally classified as well-differentiated (i.e., grade 1 and grade 2). The occurrence of central nervous system metastases in NETs presents a dual challenge, both of which are related to their low recurrence rate, which limits the chance for conclusive investigations, and the importance of the anatomical district involved, which restricts PRRT or radionuclide therapy with lutetium-177 oxodotreotide [(^177^Lu)-DOTATATE]. This issue was illustrated by Okwundu et al. in a dedicated case report. The patient presenting with type 3 well-differentiated gastric NET had widespread metastatic disease, including central nervous system lesions in the pineal gland and left cerebellopontine angle (CPA). After four doses of 7.4 GBq of [^177^Lu]-DOTATATE, local control of the pineal and CPA lesions was achieved for 23 months. However, CNS involvement was confirmed as a poor prognostic factor, which explains the subsequent development of diffuse leptomeningeal progression.

Transarterial radioembolization or TARE for the treatment of hepatocellular carcinoma (HCC) represents another “smart” way of using radionuclides for therapeutic purposes. In this regard, Gelardi et al. aimed to assess the role of albumin-bilirubin (ALBI) grade in predicting patient outcome, compared to HCC extent and liver function according to the Child-Pugh (C-P) classification.

In particular, Barcelona Clinic Liver Cancer (BCLC), C-P, and ALBI scores were retrospectively established at the time of TARE in 72 patients and correlated with OS and PFS. As expected, ALBI grade allowed an effective stratification into risk groups of patients with intermediate-advanced HCC undergoing TARE, although not in all subsets this was statistically relevant, since disease burden and the degree of portal vein thrombosis (PVT) independently impact the outcome of patients.

Last but not least, the present Research Topic would not have been complete without a dedicated review of the application of radionuclide therapy in breast cancer, which was thoroughly illustrated by Musket et al. The authors addressed both current and future highlighting of radionuclide therapy, emphasizing the need for correlative biomarker studies to draw meaningful conclusions for individualizing critical therapeutic decisions beyond the setting of clinical trials.

In conclusion, despite the long-standing biases and gender stereotypes, women in radionuclide therapy have played and continue to play a vital role in driving innovation and progress in medicine (1, 4). The highly specialized field of radionuclide therapy is not an exception. Their contributions, along with advancements in technology and research, are shaping the future of healthcare by offering new possibilities for personalized and effective treatment strategies.
